# IL-6 Regulates Mcl-_1L_ Expression through the JAK/PI3K/Akt/CREB Signaling Pathway in Hepatocytes: Implication of an Anti-Apoptotic Role during Liver Regeneration

**DOI:** 10.1371/journal.pone.0066268

**Published:** 2013-06-25

**Authors:** Chia-Hung Chou, Shuo-Lun Lai, Chiung-Nien Chen, Po-Huang Lee, Fu-Chuo Peng, Min-Liang Kuo, Hong-Shiee Lai

**Affiliations:** 1 Department of Surgery, National Taiwan University Hospital and National Taiwan University College of Medicine, Taipei, Taiwan; 2 Graduate Institute of Toxicology, National Taiwan University College of Medicine, Taipei, Taiwan; Virginia Commonwealth University, United States of America

## Abstract

**Aims:**

To investigate the role and the regulation of the long variant of myeloid cell leukemia-1 protein (Mcl-1_L_) during liver regeneration.

**Background:**

Liver regeneration is an important phenomenon after liver injury. The rat partial hepatectomy (PH) model was used to characterize liver regeneration and Mcl-1L expression after PH.

**Methods:**

Male Wistar rats were subjected to 70% PH. The expression of *mcl-1L* mRNA was determined by quantitative RT-PCR, and protein levels were analyzed by Western blot analysis and immunohistochemistry during liver regeneration. Functional evaluations of Mcl-1_L_ were tested using chemical inhibition (flavopiridol), genetic inhibition (siRNA) of Mcl-1L production, and by assaying for annexin V levels and DNA ladder formation. Serum IL-6 levels were determined by enzyme immunoassays; signal transduction of IL-6-regulated Mcl-1L expression was verified by chemical inhibitors and decoy double-stranded oligodeoxynucleotides.

**Results:**

High levels of Mcl-1_L_ were observed in remnant tissue at 4 h after PH. Administration of flavopiridol decreased Mcl-1_L_ accumulation and also inhibited liver regeneration. IL-6 administration promoted the accumulation of Mcl-1_L_ in rat hepatocytes, an effect that was impaired by siRNA treatments that reduced Mcl-1_L_ production. Chemical inhibition and decoy oligonucleotide competition demonstrated that IL-6-induced Mcl-1_L_ production required signaling mediated by JAK kinase, phosphoinositide 3-kinase (PI3K), and cAMP response-element-binding (CREB) proteins.

**Conclusion:**

Mcl-1_L_ is an anti-apoptotic protein induced during liver regeneration after PH in rats. The expression of Mcl-1_L_ is induced by IL-6 through the JAK/PI3K/Akt/CREB signaling pathway. Chemotherapy drugs that depend on Mcl-1_L_- or IL-6-related signaling should be considered carefully before use in patients undergoing hepatectomy for malignant tumor resection.

## Introduction

Liver regeneration is an important phenomenon after liver injury, and the reproducibility of the partial hepatectomy (PH) model has made it the preferred approach for studies of liver regeneration [1]. Key factors that affect liver regeneration include exogenous factors, such as pharmaceutical agents, chemicals, and nutrition, and endogenous factors, such as hormones, growth factors, angiogenic factors, anti-apoptotic factors, and factors implicated in immune reactions [2]–[5]. Many genes are turned on or are upregulated during different stages of liver regeneration, including genes related to the cell cycle, DNA replication, and mitosis [6]. However, the detailed signaling pathways of the mechanisms of liver regeneration remain unclear.

Anti-apoptotic effects are critical to liver regeneration [7]. The accumulation of Bcl-2 family members during liver regeneration suggested cell cycle-dependent regulation as well as a physiological role for apoptosis-modulating proteins during growth and proliferation [8]–[10]. Myeloid cell leukemia-1 (Mcl-1), a member of the Bcl-2 family, inhibits apoptosis by inhibiting Ca^2+^ signals within mitochondria [10]. Transcripts of the Mcl-1-encoding locus exist as two variants, which encode distinct isoforms of the Mcl-1 protein. Mcl-1_L_ (long) enhances cell survival by inhibiting apoptosis, whereas Mcl-1_S_ (short) promotes apoptosis [11]. The elimination of Mcl-1_L_ is an early and required step for DNA damage-induced apoptosis [12]. Degradation of Mcl-1_L_ is regulated by polyubiquitination, which targets Mcl-1_L_ to the proteasome pathway. Hepatocyte-specific *mcl-1* knockout mice undergo standard processes of hepatocyte-specific apoptosis [13]. Nonetheless, *mcl-1* knockout mice exhibit liver damage and increased apoptotic susceptibility of murine hepatocytes, suggesting that Mcl-1 is a crucial anti-apoptotic factor in the liver [14]. Other studies confirm that Mcl-1 and Bcl-xL cooperatively maintain the integrity of hepatocytes in developing and adult murine livers [9].

*Mcl-1* expression is tightly regulated by interleukin-6 (IL-6) [15], an important cytokine involved in liver regeneration. IL-6 is released from Kupffer cells and contributes to liver regeneration after PH. *Stat3 activation in hepatocytes occurs as a consequence of IL-6 production*. [1]. Moreover, IL-6 upregulates *mcl-1* expression through a STAT3-dependent pathway in cholangiocarcinoma cells [16]. However, the role of Mcl-1_L_ in the IL-6-related pathway during liver regeneration is not well clarified. We investigated the role of the Mcl-1_L_ anti-apoptotic protein during liver regeneration after PH in rats, including the pathway by which Mcl-1_L_ accumulation is regulated by IL-6.

## Methods

### Animals and study groups

Male Wistar rats (purchased from Charles River, Osaka, Japan) weighing approximately 200 g each were used in this study. All rats were randomly assigned to two groups that were subjected to either 70% PH or a sham operation (SO). PH then was performed through a midline laparotomy by aseptically extirpating the median and left lateral lobes, accounting for approximately 70% of the original liver, according to the procedure of Higgins and Anderson [17]. Each group of rats was further divided into nine subgroups (10 rats each) that were sacrificed either pre-operatively (0 h), 4, 6, 24, 48, or 72 hours post-operatively. At sacrifice, the remnant liver was excised and weighed. The original liver weight was estimated retrospectively based on the excised liver weight after 70% PH. For each time point, the ratio of remnant liver weight to the estimated original liver weight (RLW/OLW) was calculated as a percentage value. Part of the removed liver was embedded in paraffin and sectioned. The remaining liver tissue was prepared for q-RT-PCR and Western blot analysis. The animal study was approved by the National Taiwan University College of Medicine and College of Public Health Institutional Animal Care and Use Committee (No. 20060181).

### Determination of*mcl-1* mRNA Expression by Q-RT-PCR

The total RNA was isolated from the liver tissue using the RNAzol B reagent (Biotecx Laboratories, Houston, TX). Then cDNA was prepared from 2 µg of the total RNA with random hexamer primers (ImProm-II RT system; Promega, Southampton, UK). The level of rat *mcl-1_L_* mRNA was measured with a quantitative real-time PCR detection system (Light Cycler DNA Master SYBR Green I; Roche Molecular Biochemicals, Indianapolis, IN). The primers were 5′-CATGTTTGGCCTTCGGAGAA-3′ and 5′-GCATGTAGTTGGTGGCTGGAG-3′ for *mcl-1* and 5′-GGGAAGGTGAAGGTCGG-3′ and 5′-TGGACTCCACGACGTACTCAG-3′ for the *GAPDH* gene (used as a control). The amplification program consisted of one cycle of an initial incubation at 61°C for 20 min, followed by 40 cycles of denaturation at 95°C for 10 s, annealing at 55–57°C for 10 s, and extension at 72°C for 10 s. The amount of *mcl-1* mRNA was normalized to that of *GAPDH* mRNA and is presented in arbitrary units, with 100 corresponding to the value in the untreated control.

### Western blot analysis

Cells were lysed with lysis buffer [PBS, pH 7.4, containing 1% Nonidet P-40, 0.5% sodium deoxycholate, 0.1% sodium dodecyl sulfate (SDS), and 1× protein inhibitor cocktail (Nacalai Tesque, Kyoto, Japan). The hepatocyte lysates were centrifuged at 13,400× *g* for 10 min at 4°C. The protein concentration then was measured using the Bradford total protein assay (Bio-Rad, Hercules, CA, USA). Protein samples were loaded at 50 µg per well, separated by SDS-polyacrylamide gel electrophoresis (SDS-PAGE), transferred onto polyvinylidene difluoride membranes, and immunoblotted with various antibodies. The results were quantified by densitometry (Bio-Rad, GS-800 densitometer). Data were expressed as relative density (RD) compared to lane 1, which was defined as 1. The respective lanes were compared with lane 2 for the statistical analysis. The internal control level (as standard protein occurring naturally in these cells) was used as the reference.

### Immunohistochemical assay

The slides were rehydrated in PBS for 15 min and endogenous peroxidase was inhibited by exposure (10 min at room temperature) to a solution of 3% H_2_O_2_ in methanol. Samples were blocked (30 min at room temperature) using 5% nonfat milk in PBS. Slides then were incubated with anti-rat Mcl-1L antibody (sc-958, Santa Cruz Biotechnology, Inc.), or Ki67 (sc-7846, Santa Cruz Biotechnology, Inc.) or for the terminal deoxynucleotidyl transferase-mediated dUTP nick end labeling (TUNEL) assay (R&D Systems), for 16 hours at 4°C. The peroxidase-conjugated secondary antibody was incubated for 1 hour at room temperature, and slides were developed by immersing them in 0.06% 3,3′-diaminobenzidine tetrahydrochloride (DAKO), followed by counterstaining with Gill's Hematoxylin V.

### Effect of flavopiridol on liver regeneration

Flavopiridol is a cyclin-dependent kinase inhibitor that induces apoptosis of leukemic cells by inhibiting *mcl-1_L_* expression [18]. This compound was used to downregulate *mcl-1_L_* expression in the 70% PH model. The rats were randomly assigned to control and treatment groups (three each). The treatment rats received 2.5 mg/kg of flavopiridol (Sigma Co., St. Louis, MO, USA), and the control rats received vehicle (0.1% DMSO in saline) by intraperitoneal injection. PH was performed 24 h after flavopiridol or vehicle administration. The rats were then sacrificed and the remnant liver tissues were taken either pre-operatively (0 h), or at 4, 6, 24, 48, or 72 hours post-operatively.

### Cell culture of rat hepatocytes

Primary cultures of rat hepatocytes were obtained from regenerating rat livers, specifically from remnant liver specimens recovered 24 h after PH. The liver cell suspensions were combined with 10 ml of Ficoll-Paque (GE Healthcare, Stockholm, Sweden) and centrifuged at 450× *g* for 15 minutes, and the cells in interphase were then collected. The cells were treated with 80 IU/ml hyaluronidase in 1 ml of human tubal fluid medium (Irvine Scientific, Santa Ana, CA, USA) for 10 minutes. The cells were washed and suspended in RPMI-1640 medium (Gibco, Grand Island, NY, USA) containing 10% fetal bovine serum (FBS), 100 U/ml penicillin, 0.1 mg/ml streptomycin, and 0.25 mg/ml amphotericin. The cells then were incubated at 37°C in a humidified 5% CO_2_ atmosphere. The isolated rat hepatocytes were cultured for 2 passages before following experiments.

### Measurement of serum IL-6 levels by enzyme-linked immunosorbent assay ( ELISA)

The serum levels of IL-6 were determined by enzyme immunoassay (R&D Systems, Minneapolis, MN, USA) at different time points after PH.

### Annexin V assay

1×10^5^ cells were collected, washed, and resuspended in 100 µl of annexin V binding buffer [10 mM HEPES (pH 7.4), 140 mM NaCl, and 2.5 mM CaCl_2_]. Following addition of fluorescein isothiocyanate-conjugated annexin V (5 µl; Invitrogen Corporation, Carlsbad, CA, USA) and propidium iodide (1 µl, 1 mg/ml; Invitrogen), the cells were incubated for 20 min. After incubation, annexin V binding buffer (400 µl) was added, and the cells were analyzed by flow cytometry (Caliber, BD Biosciences, San Jose, CA, USA). The cell populations positive for annexin V and negative for propidium iodide were regarded as apoptotic. Data were quantified using CellQuest software (BD Immunocytometry Systems, San Jose, CA, USA).

### DNA ladder analysis

1×10^6^ cells/dish were collected and DNA was extracted by the phenol-chloroform method. The DNA electrophoresis proceeded in 2% agarose gels in Tris/acetate acid/EDTA buffer. The DNA fragments were visualized by staining with ethidium bromide and digital photography.

### Inhibition of Mcl-lL expression by siRNA

Rat hepatocytes were transfected with human-specific *mcl-1L* siRNA (sc-43912) or control siRNA (sc-37007) (25 nM) in serum-free Opti-MEM by the Oligofectamine method (Invitrogen Corporation, Carlsbad, CA, USA) for 24 hours.

### Chemical inhibitors

Specific chemical inhibitors of JAK (420097 InSolution™ JAK Inhibitor I) was purchased from EMD Millipore Chemicals, PI3K/Akt (LY294002), MAPK/ERK (PD98059), and PKC (staurosporine) kinases were purchased from Sigma Company (St. Louis, MO, USA).

### Double-stranded oligodeoxynucleotide decoys

This study used synthetic double-stranded oligodeoxynucleotides (ODNs) as ‘decoy’ *cis* elements to block the binding of nuclear factors to promoter regions of targeted genes, thus, inhibiting gene transactivation. The following sequences of phosphorothioate ODNs were used: NF-κB decoy ODN, 5′-CCTTGAAGGGATTTCCCTCC-3′; CRE decoy ODN, 5′-TGACGTCATGACGTCATGACGTCA-3′; control decoy ODN, 5′-TTGCCGTACCTGACTTAGCC-3′. For transfection of hepatocytes, the decoy or scrambled decoy was mixed with Transfast transfection reagent (Promega, Madison, WI, USA) for 15 minutes and then was incubated with the cells in serum-free medium.

### Statistical analysis

In this study, each experiment was repeated at least three times on different occasions. Data were presented as mean ± SD. The data were examined with one-way ANOVA, followed by Tukey test for multiple comparisons. Significance level was set as *P*<0.05 by two-tailed test.

## Results

### Time-dependent regeneration of liver following PH

**Figure 1:**
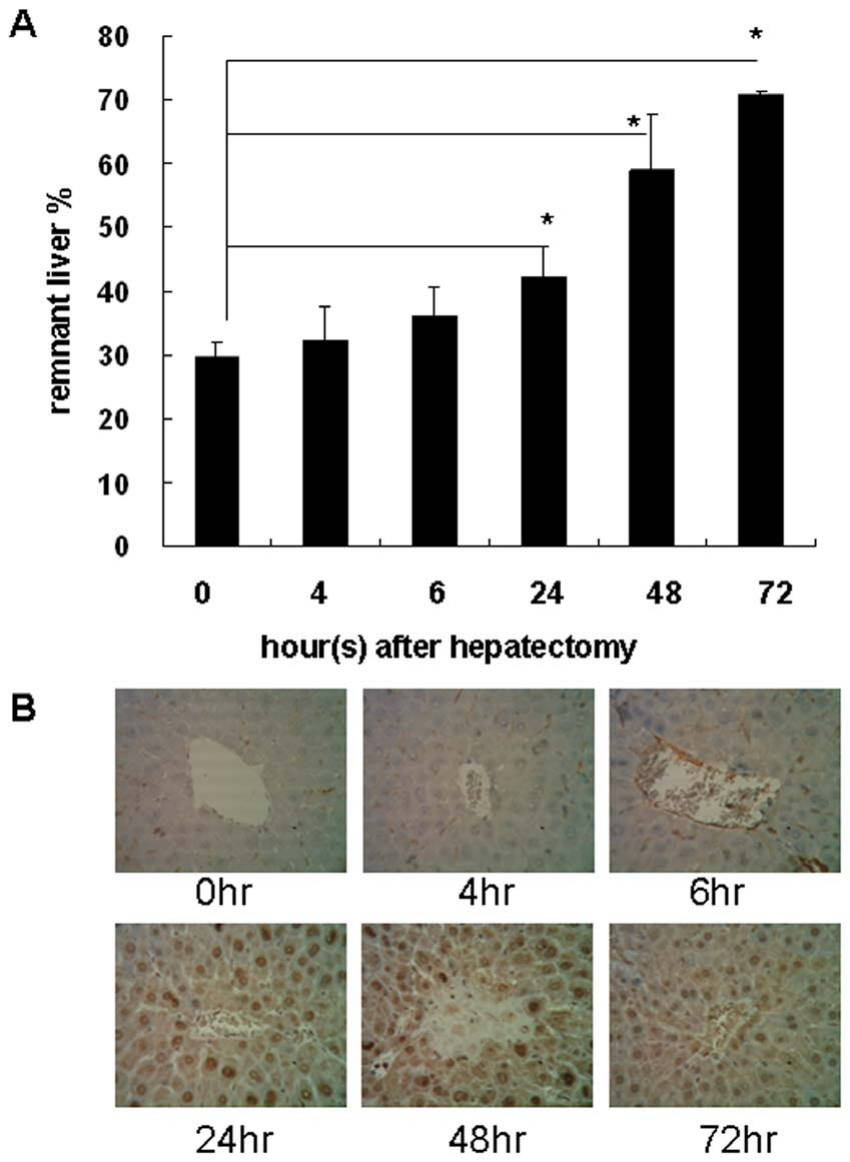
Changes in the ratio of remnant liver weight to original liver weight (RLW/OLW) after 70% partial hepatectomy (PH). (A) OLW was estimated retrospectively from the excised liver weight after 70% PH. Data are presented as mean ± S.D., and comparisons were made between groups as indicated. *P<0.05. (B) ki67 staining of remnant liver tissue. Magnification, 400×.

The percentage of RLW/OLW was 29.8±2.3% after PH. The percentage significantly increased to 42.3±4.7%, 58.9±9%, and 71.1±0.5% at 24, 48, and 72 h after PH, respectively (Figure 1A). Meanwhile, staining for the ki67 cell proliferation marker revealed that hepatocytes were obviously proliferating at 24, 48 and 72 hours after PH (Figure 1B).

### Mcl-1_L_ accumulation after PH

**Figure 2:**
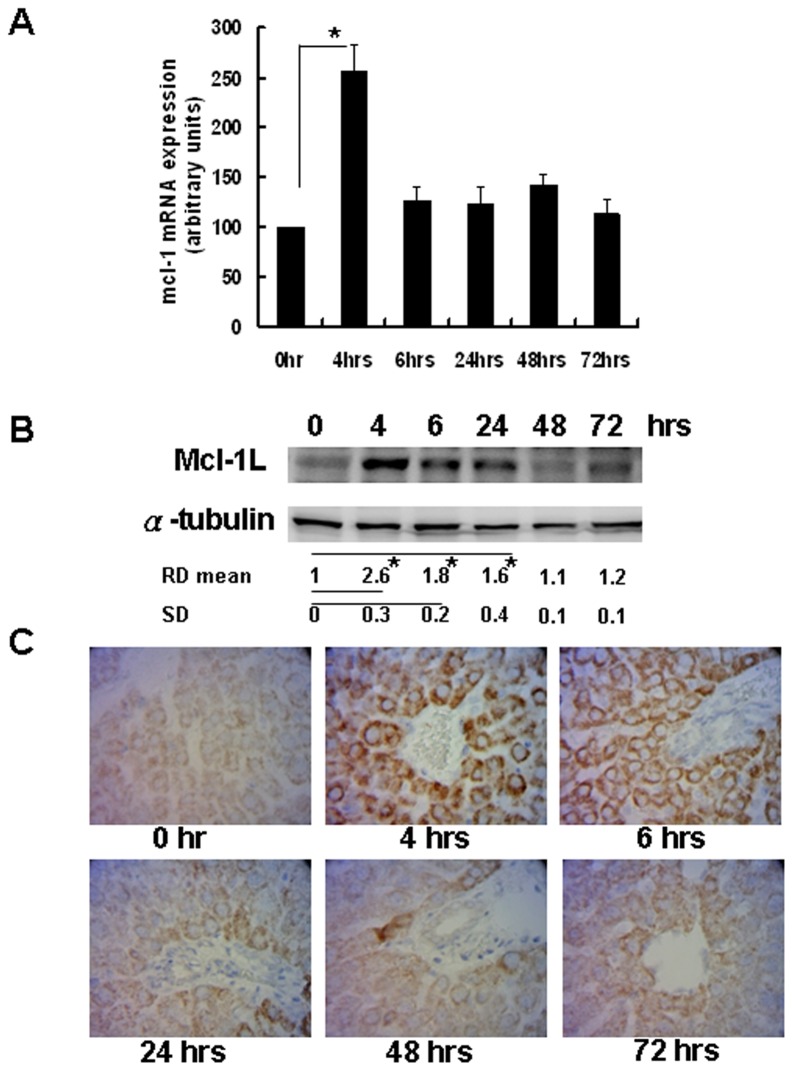
mRNA and protein expression of Mcl-1L during liver regeneration. Remnant liver tissue from the indicated time points was used to determine (A) mcl-1 mRNA expression by q-RT-PCR; *P<0.05 and (B) protein levels of Mcl-1L as detected by Western blotting. (C) Parafilm sections of the remnant liver were used for Mcl-1 detection by IHC. Magnification, 400×.

Evaluation by q-RT-PCR revealed that *mcl-1* mRNA expression was significantly increased 4 hours after PH (Figure 2A). Analysis of Mcl-1L protein levels by Western blotting revealed that Mcl-1L was expressed at 4 and 6 hours and decreased at 24 to 72 hours after PH (Figure 2B). As shown by immunohistochemical staining, Mcl-1_L_ accumulated in the cytoplasm of hepatocytes, with the highest levels observed at 4 and 6 hours after PH (Figure 2C).

### Mcl-1L expression is required for rat liver regeneration

**Figure 3:**
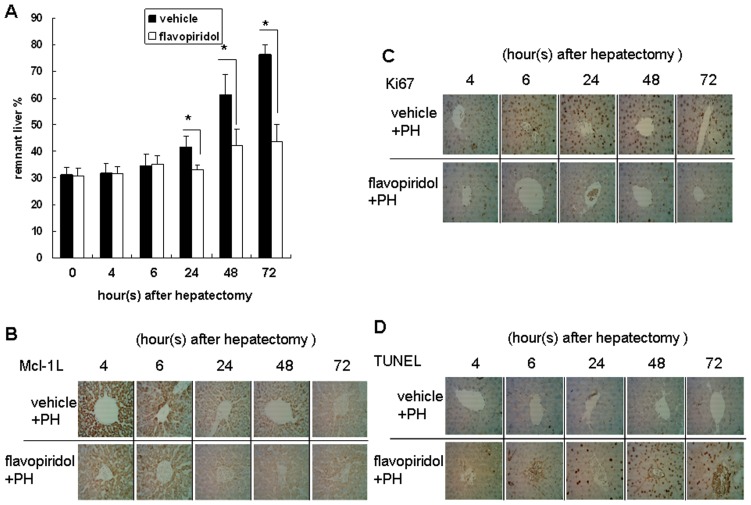
Mcl-1L chemical inhibitor flavopiridol inhibits liver regeneration in rats. (A) Rats were pretreated with flavopiridol (2.5 mg/kg) or vehicle (control) 24 hours before partial hepatectomy (PH). (A) The remnant liver weight (RLW) of each group was measured at the indicated time periods after PH and expressed as a percentage of the original liver weight (OLW). Data are compared between the vehicle and flavopiridol-treated groups. *P<0.05. (B) The expression of Mcl-1L in remnant liver tissue was determined by IHC. (C) ki67 staining. (D) TUNEL staining. Magnification, 400×.

To clarify the role of Mcl-1L in rat liver regeneration, an Mcl-1L-specific chemical inhibitor, flavopiridol [17], was used to inhibit Mcl-1L expression. Rats were pretreated with or without flavopiridol for 24 hours prior to PH. Liver regeneration was significantly inhibited in the flavopiridol-treated group. The percentages of remnant livers in vehicle and flavopiridol-treated groups after PH were 41.6±4% vs. 33.1±1.7%, 61.2±7.8% vs. 42.3±5.9%, and 76.4±3.8% vs. 43.8±6.2% at 24 h, 48 h, and 72 h, respectively (Figure 3A). The result revealed that the decrease in liver size gain happens longer to recover. Thus, the RLW/OLW percentage was significantly inhibited by flavopiridol pretreatment. Furthermore, the expression of MCL-1L was analyzed by immunohistochemistry (IHC) (Figure 3B). Furthermore, the proliferation of hepatocytes was analyzed by ki67 staining. The results revealed that cell proliferation was markedly reduced after pretreatment with flavopiridol (Figure 3C). In addition, TUNEL staining of hepatocytes (Figure 3D) revealed that pretreatment with flavopiridol induced apoptosis in the remnant liver tissue. Thus, downregulation of Mcl-1_L_ expression was associated with hepatocyte apoptosis and with the inhibition of liver regeneration.

### Elevation of serum IL-6 levels following PH

**Figure 4:**
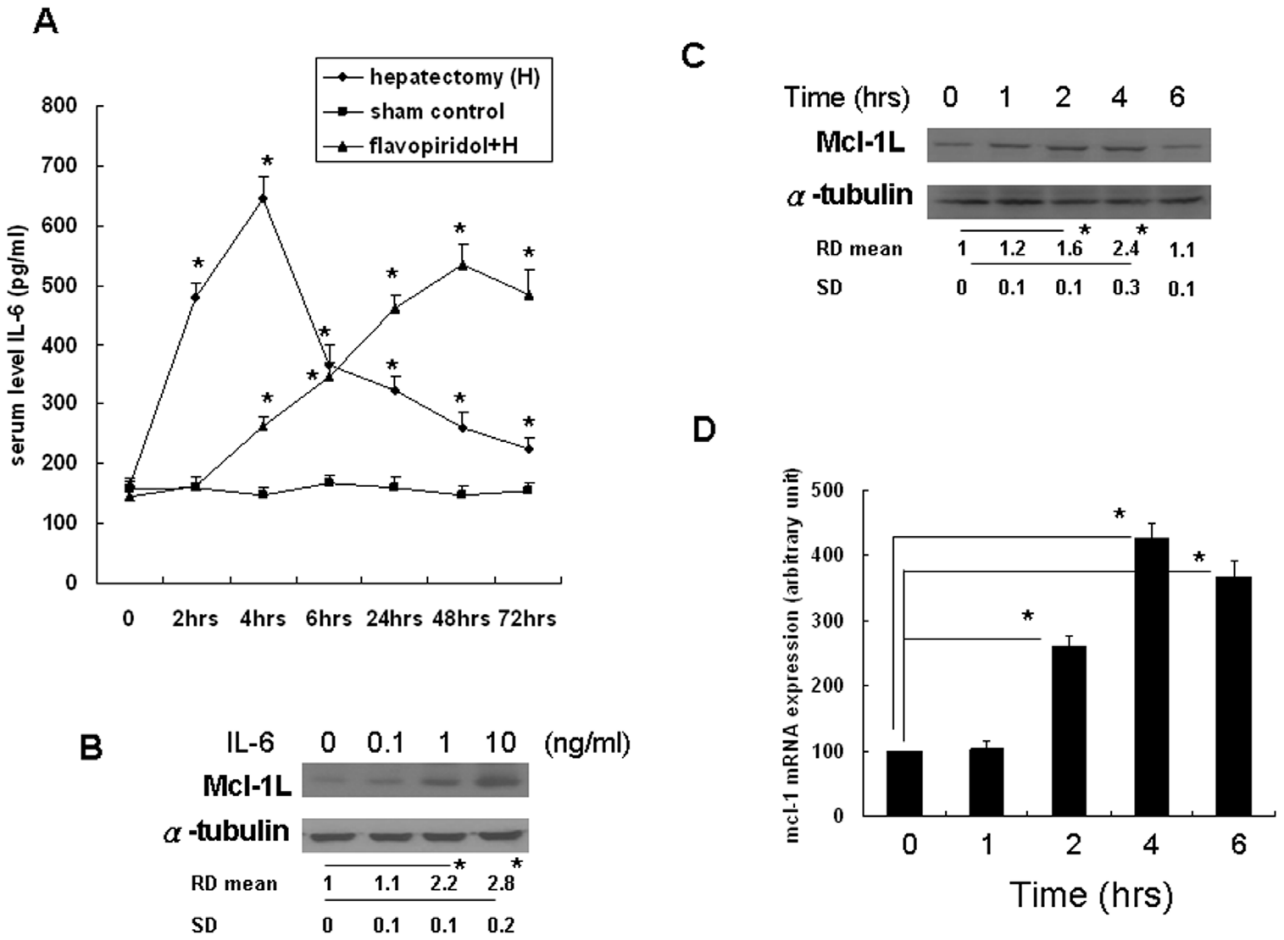
IL-6 induced Mcl-1L expression in rat hepatocytes. (A) Serum levels of IL-6 in PH rats or flavopiridol (2.5 mg/kg) treated for 24 hours before PH group or control group were determined by EIA. Data are provided as mean +/− S.D. *P<0.05. (B) Rat hepatocytes were serum-starved for 24 hours prior treated with recombinant rat IL-6 (0.1, 1 and 10 ng/ml), under serum-starved conditions, and after 4 hours, Mcl-1L protein levels were determined by Western blot analysis. (C) Rat hepatocytes were serum-starved for 24 hours prior treated with recombinant rat IL-6 ( 1 ng/ml), under serum-starved conditions, at the indicated time periods, Mcl-1L protein levels were determined by Western blot analysis. (D) Rat hepatocytes were treated with rat recombinant IL-6 (10 ng/ml) at the indicated time periods. Mcl-1 mRNA expression in rat hepatocytes was determined by q-RT-PCR. *P<0.05.

IL-6 has been found to regulate Mcl-1L expression in several types of cells, and therefore we verified the serum levels in PH rats. The mean serum level of IL-6 was 162±13 pg/ml before PH. IL-6 serum levels increased following PH, peaking at 645±37 pg/ml by 4 h before subsequently decreasing. However, levels at 72 h after PH (226±18 pg/ml) remained elevated compared to pre-PH values (Figure 4A). Serum IL-6 levels were significantly higher than in the SO controls at 2 to 48 h after PH. Meanwhile, the serum levels IL-6 in the flavopiridol treated rats was showed (Figure 4A). IL-6 serum levels did not increased following PH, peaking at 533±36.2 pg/ml by 48 h before subsequently decreasing. However, the increased expression of IL-6 in 24 to 48 hours did not correlated with the expression of Mcl-1L that showed in Figure3B.

We further investigated the induction of Mcl-1L by IL-6 in primary culture of rat hepatocytes. Previously study revealed that upon IL-6 treatment, Mcl-1 was rapidly up-regulated peaking at 4–8 h in human cancer cells [30]. Dose effect of recombinant rat IL-6 on Mcl-1L protein expression was evaluated by treating rat hepatocytes with recombinant rat IL-6 for 0.1, 1 and 10 ng/ml. The Mcl-1L protein level was determined at 4 hours. The result revealed that 1 and 10 ng/ml of recombinant rat IL-6 significantly induced Mcl-1L protein expression in a dose-dependent manner (Figure 4B). Furthermore, 1 ng/ml of recombinant rat IL-6 was used to evaluate the time course of Mcl-1L protein expression. The result revealed that 1 ng/ml of recombinant rat IL-6 significantly induced Mcl-1L protein expression at 2 and 4 hours, the result revealed that recombinant rat IL-6 induced Mcl-1L protein expression in a time-dependent manner (Figure 4C). 10 ng/ml of recombinant rat IL-6 was used to treat rat hepatocytes, the transcriptional regulation of mcl-1 mRNA was determined by Q-RT-PCR. The result revealed that IL-6 significantly increased mcl-1 mRNA expression at 2, 4 and 6 hours (Figure 4D).

### IL-6-mediated Mcl-1_L_ accumulation is dependent on the JAK/PI3K/Akt/CREB signal transduction pathway

**Figure 5:**
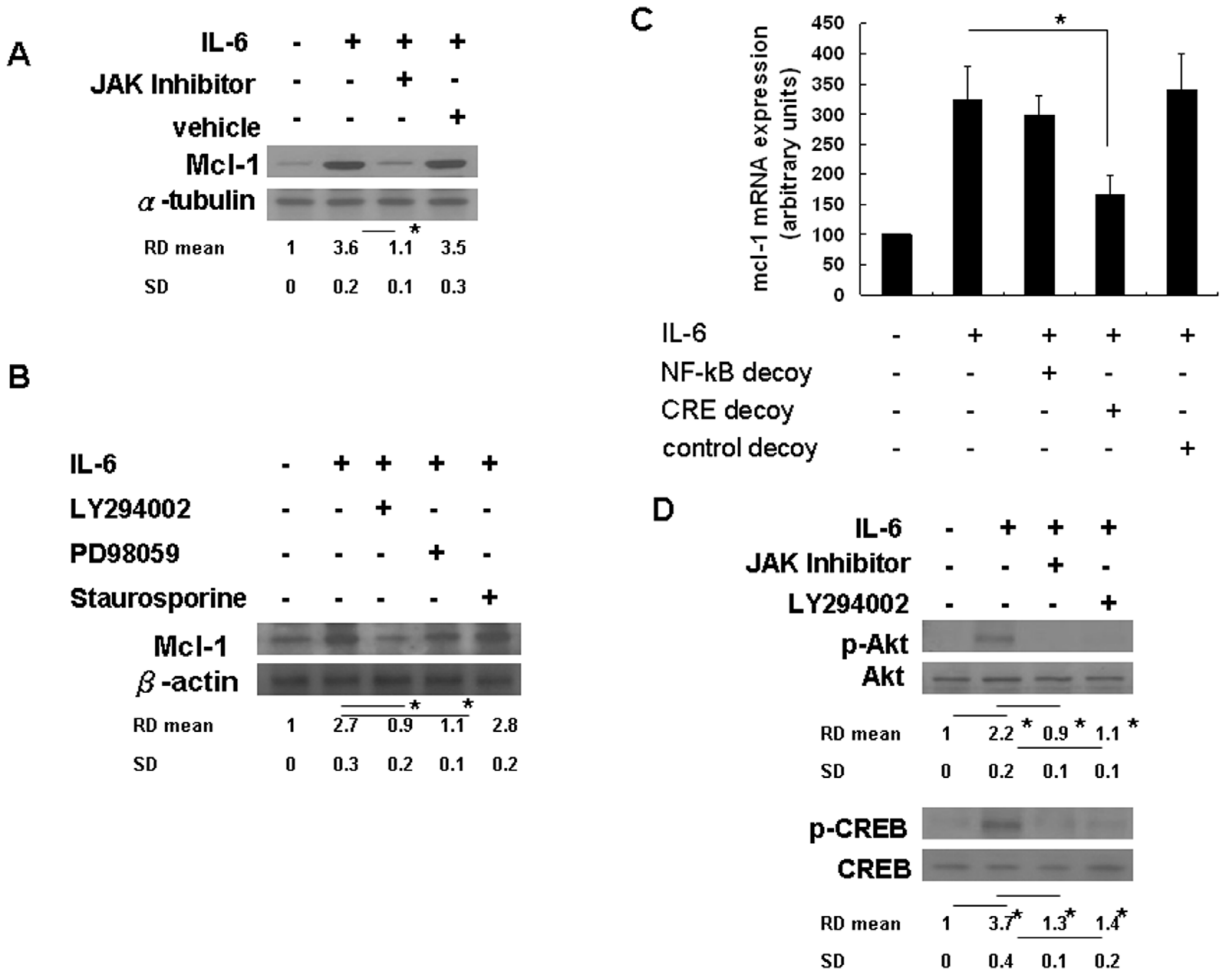
JAK/PI3K/Akt/CREB signaling is involved in IL-6-induced Mcl-1L expression in hepatocytes. (A) Rat hepatocytes were pretreated with chemical inhibitors, JAK Inhibitor InSolution™ 420097 (20 nM), for 1 h prior to IL-6 (10 ng/ml) treatment. After 4 h, the Mcl-1L protein levels were determined by Western blotting. Between-group comparisons are as indicated with *p<0.05. (B) Rat hepatocytes were pretreated with chemical inhibitors, LY294002 (50 µg/mL), PD98059 (50 µg/mL), and staurosporine (20 nM) for 1 h prior to IL-6 (10 ng/ml) treatment. After 4 h, Mcl-1L expression was analyzed by Western blotting. Between-group comparisons are as indicated. *p<0.05. (C) Rat hepatocytes were pretreated with the NF-κB and CREB decoy ODN (10 µM) for 24 h prior to IL-6 treatment. After 4 h, mcl-1 mRNA expression was analyzed by q-RT-PCR. Data are fold-induction relative to the control (Lane 1). Between-group comparisons are as indicated. *p<0.05. (D) Rat hepatocytes were pretreated with JAK Inhibitor InSolution™ 420097 (20 nM), or LY294002 (50 µg/mL) for 1 h prior to IL-6 treatment. After 30 minutes, p-Akt, Akt, p-CREB and CREB were determined by Western blotting. Between-group comparisons are as indicated. *p<0.05.

Previous reports on IL-6 signaling showed that binding of IL-6 to the α-subunit of its receptor (IL-6R) triggered the recruitment of gp130, which subsequently activated downstream signaling pathways, including JAK/STAT3, PI3K, MAPK, and PKC [18]. We confirmed the involvement of the JAK pathway using the JAK Inhibitor InSolution™ 420097. As shown by Western blot and quantitative analyses, IL-6 induction of Mcl-1_L_ accumulation was attenuated by JAK Inhibitor (Figure 5A). Using the same experimental method, we also tested the role of MAPK, PKC, and PI3K signaling by use of the chemical inhibitors PD98059, staurosporine, and LY294002, respectively. Our results demonstrate that IL-6-induced Mcl-1L accumulation was significantly reduced by treatment with LY294002 but not by PD98059 or staurosporine (Figure 5B).

The promoter sequences upstream of *mcl-1* contain conserved putative binding sites for nuclear factor-κB (NF-κB) and cAMP response-element (CRE) binding protein (CREB). We, therefore, investigated the role of NF-κB and CREB in IL-6-induced *mcl-1* gene expression using synthetic double-stranded ODNs as decoy *cis* elements. The CRE decoy, but not the NF-κB decoy, significantly reduced IL-6-induced *mcl-1* transcript levels (Figure 5C).

We next used JAK Inhibitor InSolution™ 420097 and LY294002 to evaluate the cascade between JAK and PI3K by measuring the phosphorylation of Akt, which is a downstream effector of PI3K. IL-6-induced Akt phosphorylation (measured as RD of p-Akt/Akt) was significantly reduced following treatment with JAK Inhibitor, suggesting that PI3K is a downstream target of JAK (Figure 5D, upper panel). Meanwhile, IL-6-induced CREB phosphorylation (measured as RD of p-CREB/CREB) was significantly reduced in the presence of JAK Inhibitor and LY294002, suggesting that CREB was a downstream target of JAK and PI3K (Figure 5D, lower panel). Taken together, these results indicate that IL-6 induced Mcl-1L expression through the JAK/PI3K-mediated CREB activation pathway in rat hepatocytes.

### IL-6-mediated Mcl-1L expression plays a critical role in serum starvation-induced apoptosis in hepatocytes

**Figure 6:**
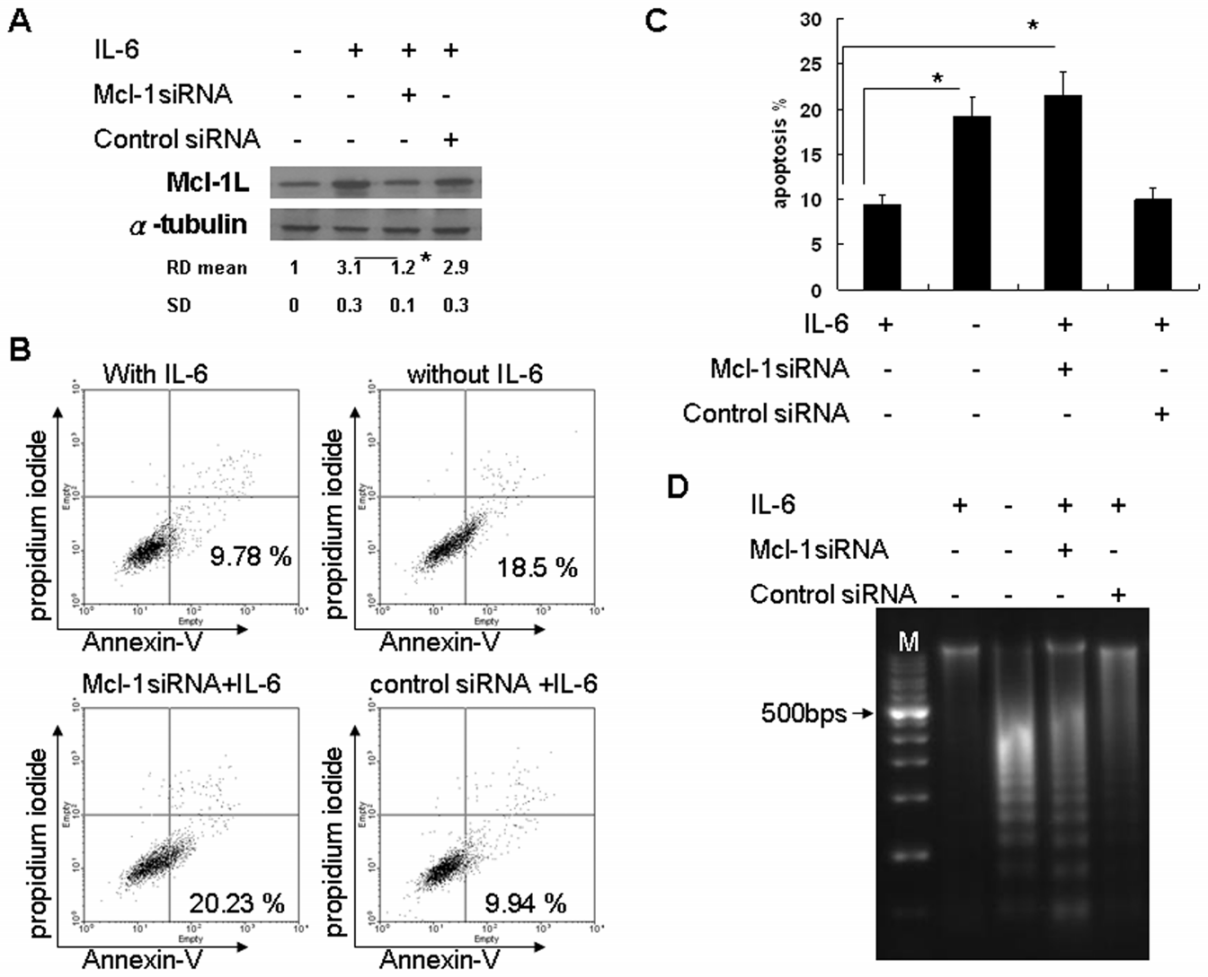
IL-6 induced Mcl-1L expression plays an anti-apoptotic role in hepatocytes. (A) Rat hepatocytes were pretreated with mcl-1L siRNA (25 nM) or control siRNA (25 nM) for 24 h prior to IL-6 (10 ng/ml) treatment; four hours later, Mcl-1L levels were determined by Western blot analysis. Between-group comparisons are as indicated. *p<0.05. (B) Rat hepatocytes were pretreated with mcl-1L siRNA or control siRNA for 24 h prior to IL-6 treatment or not in serum-free conditions for another 48 hours. The percentage of apoptotic hepatocytes were determined by annexin-V staining. The illustration shown represents one of three independent experiments. (C) Quantitative result of annexin-V staining. Between-group comparisons are as indicated. *p<0.05. (D) Similar treatment protocol as described in (C), another 72 hours. DNA ladder analysis was performed. The illustration shown is representative of three independent experiments.

To confirm the anti-apoptotic role of Mcl-1L, an siRNA strategy was used to abolish the expression of Mcl-1L in rat hepatocytes. The induction of Mcl-1L was significantly inhibited by *mcl-1* siRNA, but not by control siRNA (Figure 6A). The percentage of apoptotic hepatocytes pretreated with *mcl-1* siRNA or recombinant IL-6 was detected by annexin-V staining (Figure 6B). Quantitative analysis revealed that recombinant rat IL-6 significantly rescued serum starvation-induced apoptosis. Meanwhile, *mcl-1* siRNA significantly inhibited the rescue effect of recombinant rat IL-6 in rat hepatocytes (Figure 6C). We also confirmed the apoptosis phenomena by DNA ladder analysis (Figure 6D), as the results revealed that recombinant rat IL-6 inhibited DNA fragmentation of serum-starved rat hepatocytes compared with those without IL-6 treatment. The *mcl-1* siRNA, but not the control siRNA, induced DNA fragmentation. Therefore, Mcl-1L played an important role in the anti-apoptotic effects of IL-6.

## Discussion

Liver regeneration is one of the most important basic mechanisms by which the liver responds to a loss of liver mass or liver injury, such as PH. However, the signaling mechanisms underlying liver regeneration are poorly understood. In this study, we describe the key anti-apoptotic role of Mcl-1_L_ during liver regeneration after PH, and demonstrate that Mcl-1_L_ accumulation is stimulated by IL-6 through the JAK/PI3K/Akt/CREB signaling pathway. The anti-apoptotic role of Mcl-1_L_ is well defined in several types of cancer cells [19],[20],[21]. It is reported that apoptosis of hepatocytes is increased in mice with hepatocyte-specific deletion of *mcl-1* [13]. Our results showed that Mcl-1_L_ accumulated in the cytosol within the first 4 h after surgical PH in rats. Using Western blot analysis, we showed that the Mcl-1_L_ accumulation was significantly reduced by flavopiridol, consistent with the reduced cell proliferation observed in flavopiridol-treated animals. Our data suggested that the anti-apoptotic effect was an important step in the process of liver regeneration after PH, and that Mcl-1_L_ was required for this process.

Oncostatin M is a member of the interleukin (IL)-6 cytokine family, oncostatin M has been shown to induce maturation of mouse hepatocytes derived from embryonic day 14.5 liver, Tetsuhiro Hamada and colleagues examined whether the introduction of oncostatin M cDNA enhances the regeneration of livers damaged by dimethylnitrosamine in rats. The results revealed that oncostatin M is an important mediator for proliferation and anti-apoptosis of hepatocytes [22]. Furthermore, histological examination showed that oncostatin M gene therapy reduced centrilobular necrosis and inflammatory cell infiltration and augmented hepatocyte proliferation [23]. The *pim-3* gene also protected rats from fulminant hepatic failure by inhibiting liver apoptosis and improving the inflammatory response of liver tissues, which was associated with inhibiting the expression of inflammatory mediators and promoting the production of the anti-apoptosis protein Bcl-2 [24]. Liver cell death through apoptosis is a key pathogenic feature of acute and chronic liver diseases, although controlled hepatocyte apoptosis is essential for liver homeostasis. Mcl-1 is a member of the Bcl-2 protein family, and deletion of the *mcl-1* gene can result in life-threatening liver phenotypes due to apoptotic induction [25]. We believe that therapeutic strategies to enhance anti-apoptotic effects are feasible, as preventive and curative means, for the treatment of patients with liver damage.

We found a significant increase in the serum IL-6 levels from 2 to 4 h after PH. IL-6 is critically involved in liver regeneration after PH [1]. Cells of bone marrow origin, most likely Kupffer cells, regulate the regenerative capacity of the hepatocytes through IL-6 expression [26]. IL-6 *trans*-signaling via soluble IL-6R also is important for the physiologic response of the liver to CCl_4_-induced chemical damage [27]. Platelets promote the proliferation of liver-specific endothelial cells (LSECs) and induce the production of IL-6 and vascular endothelial growth factor (VEGF). IL-6 from LSECs induces the proliferation of parenchymal hepatocytes [28].

We evaluated the anti-apoptotic role of IL-6 in rat and human hepatocytes with recombinant IL-6 proteins, and our results showed that IL-6 did have an anti-apoptotic function in hepatocytes. We further investigated the role of IL-6-induced Mcl-1_L_ accumulation in hepatocytes using gene knockdown via an siRNA strategy. The results revealed that Mcl-1_L_ mediated the anti-apoptotic effects of IL-6. In the presence of soluble IL6-R, IL-6 expression acts through Mcl-1 to rescue endothelial cells from radiation-induced death [29]. In human oral squamous carcinoma cells, Mcl-1L protein was present in radioresistant sublines generated by fractionated ionizing radiation [20].

Concomitant activation of the PI3K/Akt and the STAT3 pathways mediates the anti-apoptotic effects of IL-6 against TGF-beta, with the STAT3 pathway likely playing a major role [30]. Realizing that signaling transduction pathways are important for developing molecular targeting strategies, we used RHCs to evaluate the signal transduction pathways involved in IL-6-induced Mcl-1 accumulation in normal hepatocytes. IL-6 signals via two different pathways: classic signaling via the membrane-bound IL-6R, and IL-6 *trans*-signaling via a naturally occurring soluble IL-6R (sIL-6R) [31]. The second pathway widens the scope of IL-6 signaling because cells lacking membrane-bound IL-6R nonetheless can be stimulated by the *trans*-signaling pathway. Galun E et al. (2000) demonstrated that a designer molecule, Hyper IL-6, could serve as an agonist of sIL-6R; by mimicking IL-6 *trans*-signaling, Hyper-IL-6 accelerated liver regeneration [37]. Another designer molecule, sgp130Fc, specifically blocked IL-6 *trans*-signaling. These proteins can be used to investigate the contribution of IL-6 classic and *trans*-signaling to liver damage and regeneration.

In the present study, we showed that recombinant IL-6 directly interacted with hepatocytes, and that the JAK/PI3K/Akt/CREB signaling pathway was involved in Mcl-1_L_ regulation. IL-6 also stimulates the proliferation of primary cultured chicken hepatocytes through the PKC, p44/42 MAPKs, and PPARdelta pathways [32]. Sudo K and colleagues found that TNF-alpha KO- and IL-6 KO-transplanted mice compared with WT-transplanted mice showed decreased hepatocyte DNA synthesis after PH. Their results revealed that TNF-alpha KO-transplanted mice showed no nuclear factor kappa B (NF-kappaB) and signal transducer and activator of transcription (STAT) 3 binding after PH, while IL-6 KO-transplanted mice showed NF-kappaB, but not STAT3, binding. Lack of AP-1 or C/EBP binding or expression of c-jun or c-myc mRNA after PH was unrelated to the timing and amount of DNA replication. They concluded that the TNF-alpha and IL-6 signals from the blood are necessary for liver regeneration and NF-kappaB and STAT3 binding are activated via TNF-alpha and IL-6 signal pathways [33]. In HCC cells, it was found that IL-6 upregulates Mcl-1 through a PI3K/Akt-dependent pathway [30]. Clinically, it is suggested that caspase activation and apoptosis are involved in acute liver failure of patients with spontaneous recovery; caspase-independent cell death could be more relevant in irreversible forms of liver failure. These findings are important for therapeutic options for acute liver failure and also suggest that measurement of caspase activation might be of prognostic value to predict the outcome of acute liver failure [34]. In this study, treatment with flavopiridol inhibited the proliferation of hepatocytes, whereas a cell proliferative marker and an apoptotic marker demonstrated that the downregulation of Mcl-1_L_ severely affected liver regeneration after PH. Our results strongly suggested that the anti-apoptotic effect was required in normal hepatocytes during liver regeneration after PH. Therapeutic drugs such as Carfilzomib [35] and Sabutoclax [36] (which target Mcl-1 or IL-6-related signaling) should be considered to avoid damage to hepatocytes and to enhance liver regeneration.

**Figure 7:**
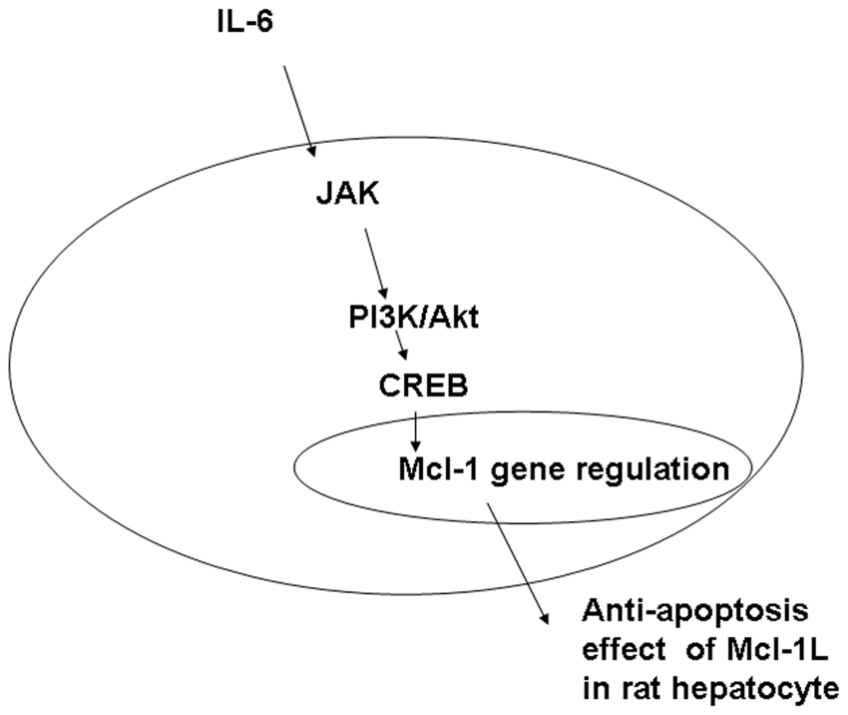
Schematic of the proposed signal transduction pathway of Mcl-1L induction in hepatocytes during liver regeneration. IL-6 regulates Mcl-1L expression through the IL-6 dependent JAK/PI3K/Akt/CREB activation pathway in rat hepatocytes.

In conclusion, our data support the hypothesis that Mcl-1_L_ is a critical, anti-apoptotic protein involved in liver regeneration after PH. We showed that IL-6 is an important inducer of Mcl-1 accumulation in hepatocytes. Furthermore, we showed that IL-6 stimulation of Mcl-1 accumulation depended on the JAK/PI3K/Akt/CREB signaling pathway in rat hepatocytes. (Figure 7).
